# Leptin: A Potential Link Between Obstructive Sleep Apnea and Obesity

**DOI:** 10.3389/fphys.2021.767318

**Published:** 2022-01-27

**Authors:** John Ciriello, Jason M. Moreau, Monica M. Caverson, Rebecca Moranis

**Affiliations:** Department of Physiology and Pharmacology, Schulich School of Medicine and Dentistry, University of Western Ontario, London, ON, Canada

**Keywords:** leptin, intermittent hypoxia, OBRb, STAT3, POMC, obesity, food intake, arcuate nucleus of the hypothalamus

## Abstract

Chronic intermittent hypoxia (CIH), a pathophysiological manifestation of obstructive sleep apnea (OSA), is strongly correlated with obesity, as patients with the disease experience weight gain while exhibiting elevated plasma levels of leptin. This study was done to determine whether a relationship may exist between CIH and obesity, and body energy balance and leptin signaling during CIH. Sprague-Dawley rats were exposed to 96 days of CIH or normoxic control conditions, and were assessed for measures of body weight, food and water intake, and food conversion efficiency. At the completion of the study leptin sensitivity, locomotor activity, fat pad mass and plasma leptin levels were determined within each group. Additionally, the hypothalamic arcuate nucleus (ARC) was isolated and assessed for changes in the expression of proteins associated with leptin receptor signaling. CIH animals were found to have reduced locomotor activity and food conversion efficiency. Additionally, the CIH group had increased food and water intake over the study period and had a higher body weight compared to normoxic controls at the end of the study. Basal plasma concentrations of leptin were significantly elevated in CIH exposed animals. To test whether a resistance to leptin may have occurred in the CIH animals due to the elevated plasma levels of leptin, an acute exogenous (ip) leptin (0.04 mg/kg carrier-free recombinant rat leptin) injection was administered to the normoxic and CIH exposed animals. Leptin injections into the normoxic controls reduced their food intake, whereas CIH animals did not alter their food intake compared to vehicle injected CIH animals. Within ARC, CIH animals had reduced protein expression of the short form of the obese (leptin) receptor (isoform OBR_100_) and showed a trend toward an elevated protein expression of the long form of obese (leptin) receptor (OBRb). In addition, pro-opiomelanocortin (POMC) protein expression was reduced, but increased expression of the phosphorylated extracellular-signal-regulated kinase 1/2 (pERK1/2) and of the suppressor of cytokine signaling 3 (SOCS3) proteins was observed in the CIH group, with little change in phosphorylated signal transducer and activator of transcription 3 (pSTAT3). Taken together, these data suggest that long-term exposure to CIH, as seen in obstructive sleep apnea, may contribute to a state of leptin resistance promoting an increase in body weight.

## Introduction

Obesity is an energy balance disorder that is affecting increasing numbers of individuals worldwide (Ogden et al., 2013; Jaacks et al., 2019). There are several variables that are currently thought to contribute to obesity including high-fat diets, low physical activity, low metabolic rates, and changes in insulin sensitivity. Obesity is associated with a positive energy balance, whereby individuals do not utilize as many calories as are consumed throughout the day (Bi et al., 2019). The caloric imbalance observed in obesity is thought to be promoted by the development of leptin resistance and is also considered to be one of the primary risk factors for both overweight and obese individuals (Myers et al., 2008, 2010; Morris et al., 2010; Crujeiras et al., 2015; Cui et al., 2017; Engin, 2017; Liu et al., 2018).

Leptin, a 16-kDa peptide hormonal product of the *obese* gene is produced and secreted by white adipose tissue in proportion to adiposity (Frederich et al., 1995; Lönnqvist et al., 1995; Caro et al., 1996; Münzberg and Morrison, 2015; Cui et al., 2017), has been shown to be elevated in obesity (Lönnqvist et al., 1995; Blevins et al., 2002; Badman and Flier, 2005; Haynes, 2005; Scarpace and Zhang, 2007; Zhang and Chua, 2017). Leptin normally plays an important role in metabolism and energy balance (Bjørbaek and Kahn, 2004; Crujeiras et al., 2015). This weight-reducing class-I cytokine is thought to signal primarily through the long form of obese (leptin) receptor (OBRb), one of the six isoforms identified for the leptin receptor (OBRa-f) (Banks et al., 2000; Myers et al., 2008; Gorska et al., 2010; Münzberg and Morrison, 2015; Zhang and Chua, 2017). When released into the circulation by adipose tissue, leptin normally acts as an important regulator of energy balance through its action on leptin receptors within the hypothalamus (Blevins et al., 2002; Badman and Flier, 2005; Scarpace and Zhang, 2007; Münzberg and Morrison, 2015; Zhang and Chua, 2017). The effects of circulating leptin are thought to be exerted primarily at the arcuate nucleus in the hypothalamus (ARC) (Satoh et al., 1997; Xu et al., 2018; Zhu et al., 2020). In the ARC, leptin inhibits hypothalamic neuronal circuits that stimulate food intake and activates circuits that increase energy expenditure, including the sympathetic nervous system (Stephens et al., 1995; Guerre-Millo, 1997; Baskin et al., 1999; Jéquier, 2002; Xu et al., 2018; Bi et al., 2019; Zhu et al., 2020). During leptin resistance the satiety and anorexigenic effects of leptin are lost, thus promoting dysregulation of caloric consumption, preventing negative feedback on energy storage sites such as the adipose tissue (Kalra et al., 1998; Crujeiras et al., 2015; Bi et al., 2019). As a result, hyperphagia persists, resulting in an increased deposition of adipose tissue mass (Lin et al., 2000). The resultant increase in adipose tissue causes a further elevation in circulating leptin concentrations, potentially exacerbating the resistance to leptin (Scarpace et al., 2002).

Hyperphagia and obesity are caused by a reduced expression of leptin signaling molecules such as the OBRb in the ARC (Sahu, 2004; Gong et al., 2008; Gorska et al., 2010; Xu et al., 2018). Once leptin binds to OBRb, phosphorylation and homodimerization of signal transducer and activator of transcription 3 (STAT3) occurs (Vaisse et al., 1996; Xu et al., 2018; Liu et al., 2021). Activation by phosphorylation of extracellular regulated kinase 1/2 (ERK1/2) also occurs (Banks et al., 2000). The translocation of pSTAT3 to the ARC neuron’s nucleus induces transcription and production of pro-opiomelanocortin (POMC) (Xu et al., 2018; Kovačević et al., 2019; Biglari et al., 2021), a major effector for satiety and energy expenditure, and suppressor of cytokine signaling 3 (SOCS3) (Banks et al., 2000; Bates et al., 2003; Münzberg et al., 2003; Liu et al., 2021), and a negative regulator of OBRb (Bjorbak et al., 2000). Another important negative regulator of leptin signaling is protein tyrosine phosphatase 1 B (PTP1B) (Sahu, 2004; Myers et al., 2008, 2010). Leptin resistance is thought to be associated with a decrease in activity of leptin within the ARC (Ladyman and Grattan, 2004; Münzberg et al., 2004, 2005; Crujeiras et al., 2015). The mechanisms that drive leptin resistance are not known but may include changes at the blood-brain barrier leptin transport system (Balland et al., 2014; Butiaeva et al., 2021), such as the short forms of the leptin receptor [OBRa (OBR_100_); Banks et al., 1999; Myers et al., 2010], at the level of the receptor (Rahmouni et al., 2008; Myers et al., 2010) or post-receptor signaling (Cheng et al., 2002; Mori et al., 2004; Myers et al., 2010), including changes in the down-stream melanocortin system (Marsh et al., 1999; Myers et al., 2010). It has recently been suggested that changes associated with Gabaergic neurons within the ARC may also contribute to obesity and possible leptin resistance (Vong et al., 2011; Zhu et al., 2020).

Obesity is now considered a major risk factor for the development of obstructive sleep apnea (OSA) (Peppard et al., 2000; de Sousa et al., 2008; Shah and Roux, 2009; Tuomilehto et al., 2013; Muscogiuri et al., 2019), a sleep-related breathing disorder characterized by nocturnal chronic intermittent hypoxia (CIH). OSA is the most common form of breathing sleep disorder (Dempsey et al., 2010) and is characterized by the repetitive cessation of respiratory airflow resulting from upper pharyngeal airway collapse and obstruction (Dempsey et al., 2010). OSA has been shown to occur in a considerable percentage of the population as it is estimated that 24 and 9% of middle-aged men and women, respectively, suffer from OSA (Young et al., 1993; Shepertycky et al., 2005), although the number of women that suffer from OSA has been shown to increase considerably after menopause (Hader et al., 2005). OSA is a pathophysiological disorder observed in normal weight (Pamidi et al., 2012), and overweight and obese individuals (Lubrano et al., 2012). The repetitive cessation of night time breathing exposes these individuals to a repeated depression of arterial partial pressure of oxygen (PO_2_) (Dempsey et al., 2010) and hypercapnia, and the decreased hemoglobin oxygen saturation results in myocardial along with systemic hypoxemia (Dempsey et al., 2010). Untreated, the initial consequences of OSA are sleepiness and an associated decrease in the quality of life resulting from the sleep fragmentation. However, if OSA is left untreated there is an increased risk of developing cardiovascular and metabolic disorders in these individuals (Haynes, 2000; Shamsuzzaman et al., 2003; Marin et al., 2005; Alam et al., 2007; Dempsey et al., 2010; Ong et al., 2013; Williams et al., 2019). Sleep disturbances associated with OSA may promote hormonal, metabolic and behavioral changes that may contribute to weight gain and the inability to lose weight (Li et al., 2006; St-Onge and Shechter, 2015; Zeng et al., 2015; Gileles-Hillel et al., 2016). Clinical evidence also suggests that OSA may have direct and long-term deleterious effects on cardiovascular structure and function through several mechanisms, including increased sympathetic nerve activity, oxidative stress, inflammation, and endothelial dysfunction (Haynes, 2000; Wolk and Somers, 2003; Wolk et al., 2003; Phillips and Cistulli, 2006; Alam et al., 2007; Dempsey et al., 2010; Ziegler et al., 2011; Drager et al., 2013). OSA has also been shown to be associated with atherosclerosis, coronary heart disease and cardiac arrhythmias (Phillips and Cistulli, 2006; Zeng et al., 2015), diabetes mellitus (Pamidi et al., 2012), and stroke and transient ischemic attacks (Yaggi et al., 2005; Dempsey et al., 2010).

Patients with OSA have also been shown to have elevated leptin levels, and a greater disposition to weight gain (Gileles-Hillel et al., 2016; Shechter, 2017; Berger and Polotsky, 2018). In an animal model of OSA, it has been found that these animals exhibit chronic intermittent hypoxia (CIH) (Fletcher et al., 1992). We have recently demonstrated in an animal model studying the effects of intermittent hypoxia that acute intermittent hypoxia alters energy balance, increases circulating plasma leptin levels (Moreau and Ciriello, 2013) regardless of body weight, and activates leptin-related signaling mechanisms within the ARC (Moreau and Ciriello, 2013). These findings suggest that OSA may influence energy intake and expenditure through effects on leptin secretion (Ong et al., 2013; Shechter, 2017; Berger and Polotsky, 2018) independent of the initial body weight. Thus, the possibility exists that there may be a reciprocal effect between OSA and body weight as previously suggested (Peppard et al., 2000; Ong et al., 2013; Gileles-Hillel et al., 2016), in that not only does obesity increase the risk of developing OSA, but in non-obese subjects with OSA, the resulting CIH from the OSA may contribute to an increase in body weight and metabolic dysfunction by further reinforcing the additional development of weight gain. To test this latter possibility in non-obese subjects, experiments were done to examine the effects of long-term exposure to CIH on body weight, food, and water intake. Additionally, as we have previously shown that CIH results in continuous elevated level of plasma leptin even though body weight is decreased initially in response to CIH (Messenger et al., 2012, 2013; Messenger and Ciriello, 2013; Moreau and Ciriello, 2013), studies were done to determine whether a possible development of a leptin resistance occurred which may contribute to an increase in body weight. Finally, the concomitant alterations of protein expression associated with leptin signaling within the ARC were investigated.

## Results

### Locomotion Changes

**FIGURE 1:**
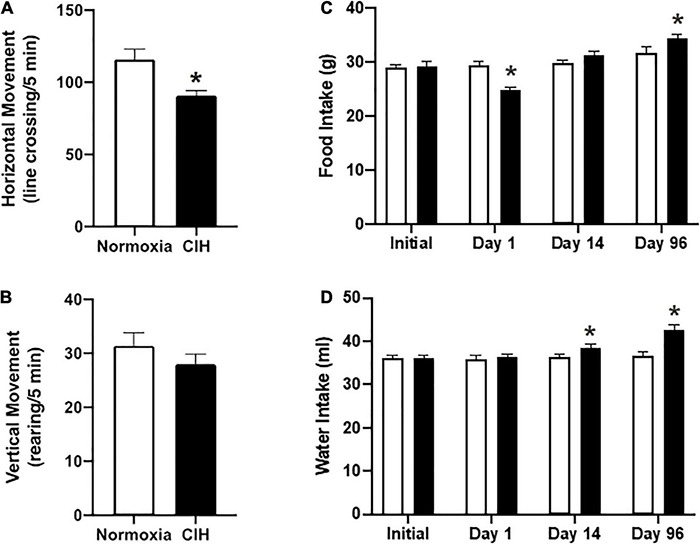
**(A,B)** Are bar charts showing both horizontal **(C)** and vertical **(D)** locomotion after CIH or normoxia exposure. Note that exposure to CIH decreased horizontal movements, although a trend toward decreased rearing was also apparent. *n* = 8 for each group. **(C,D)** Are bar charts showing food **(C)** and water intake **(D)**. Note that after the first day of CIH exposure there was a decrease in food intake, whereas by day 96, CIH induced an increase in the 24 h food intake **(C)**. In contrast, by day 14, CIH induced and increase in water intake which was maintained until the end of the study **(D)**. *n* = 16 for each group. Data are shown as mean ± standard error. *p < 0.05.

Horizontal locomotor activity was significantly reduced (*p* = 0.0228) in the CIH group compared to the normoxic controls (90.9 ± 2.5 line-crosses/5 min and 115.9 ± 7.1 line- crosses/10 min, respectively; Figure 1A). Vertical locomotor activity was not different (*p* = 0.1932) between CIH and normoxic rats (28 ± 1.9 rearing/5 min and 31.4 ± 2.4 rearing/10 min, respectively) (Figure 1B).

### Food and Water Intake

In the 16 h following daily exposure after the first day, the CIH group consumed an average of 24.9 ± 0.5 g of food that was significantly less (*p* = 0.0001) than the normoxic controls that consumed an average of 29.5 ± 0.8 g of food (Figure 1C). After 96 days of exposure, CIH animals consumed a daily average of 34.5 ± 0.6 g of food, representing an increase of approximately 18.4%, and normoxic animals consumed 31.8 ± 1.2 g of food, representing a decrease of approximately 9.4% (Figure 1C).

Both groups drank the same amount of water at the beginning of the study (CIH, 36 ± 0.9 ml; normoxic, 36.1 ± 0.7 ml) (Figure 1D). However, by the second week, the CIH group drank on average 2.3 ml more (*p* = 0.4132), and this increased amount continued until the end of the study where the CIH group drank on average 16.7% more water than the normoxic controls (CIH, 42.7 ± 1.2 ml, normoxic, 36.6 ± 0.9 ml; *p* = 0.0001) (Figure 1D).

### Food Conversion Efficiency

**FIGURE 2:**
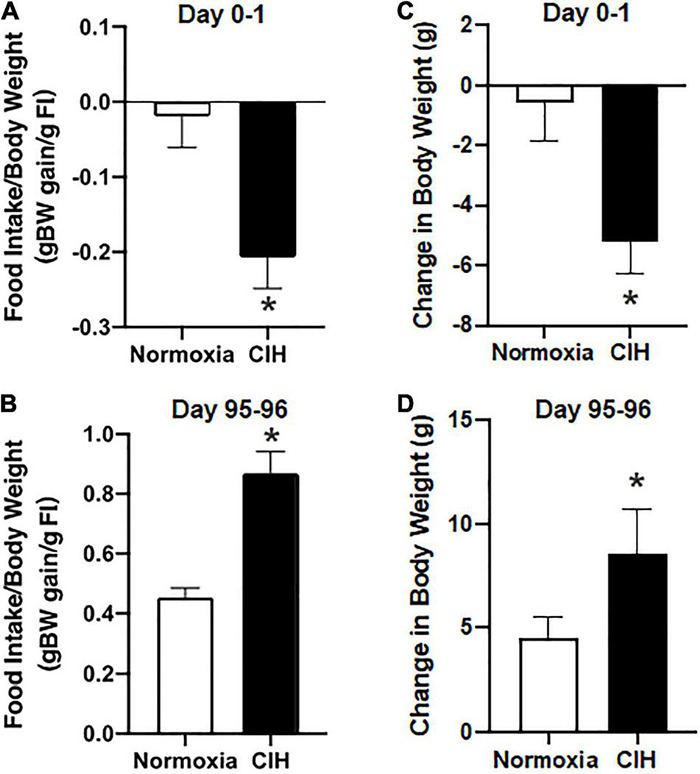
**(A,B)** Are bar charts showing food conversion efficiency after 1 day and 96-day exposure to CIH or normoxia. exposure. Note that after 1-day exposure to CIH there was a more efficient conversion of food intake, whereas after 96-days of CIH resulted in a less efficient food conversion. *n* = 16 for each group. **(C,D)** Are bar charts summarizing the 24 h body weight changes following the 8 h normoxic or CIH exposures during day 0–1 and after days 95–96. Note that immediately after exposure to CIH, there was a 24 h reduction in body weight which by day 96 was reversed in which CIH induced an increase in body weight. *n* = 16 for each group. Data are shown as mean ± standard error. *p < 0.05.

After the first day of exposure, food conversion efficiency, a measure of body weight gain compared to food intake, in the CIH group was −0.21 ± 0.04 g body weight gain/g food intake and 0.02 ± 0.04 g body weight gain/g food intake in the normoxic group (Figure 2A). After day 96, CIH animals had a food conversion efficiency of 0.87 ± 0.07 g body weight gain/g food intake, and normoxic animals had a food conversion efficiency of 0.46 ± 0.03 g body weight gain/g food intake (Figure 2B). There was an effect of exposure for CIH to increase food conversion efficiency during the first day, while the relationship of exposure length and exposure type interacted to produce a complex relationship whereby there was an increase in food conversion efficiency in the CIH animals over the 96-day exposure time, but there was a significantly reduced food conversion efficiency in normoxic animals over the same period (Figures 2A,B).

### Body Weight Changes

**FIGURE 3:**
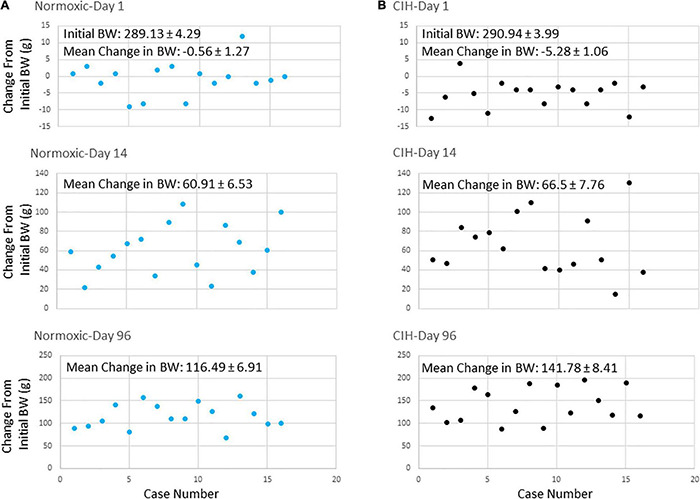
**(A,B)** Are scatter plots showing body weight changes from initial body weights at the start of the study at days 1, 14, and 96 after either normoxic (*n* = 16) or CIH (*n* = 16) exposure. Note that after 24 h exposure on day 1, body weights decreased in the CIH group **(B)** but not in the normoxic group **(A)**. By day 14, body weights were similar in both groups. However, by day 96, body weights of the CIH group were elevated compared to normoxic controls.

Prior to exposure, body weights between CIH (290.94 ± 3.99 g) (Figure 3B) and normoxic (289.13 ± 4.29 g) (Figure 3A) groups were not significantly different (*p* = 0.379). The average body weight after the first day of CIH exposure was 285.65 ± 4.56 g, while the normoxic controls weighed an average of 288.56 ± 4.66 g, were not significantly different (*p* = 0.3797) Similarly, after the first 2 weeks of exposure, CIH animals weighed 357.43 ± 7.19 g and compared to normoxic controls weighing 350.03 ± 7.13 were not significantly different (*p* = 0.3296). However, by the end of the study, those exposed to CIH weighed significantly (*p* = 0.0001) more (432.72 ± 5.72) compared to the normoxic controls (405.61 ± 6.53) (Figure 3). Contrastingly, after the first day of exposure, CIH exposed animals lost on average approximately 2% of their body weight (−5.28 ± 1.06 g), whereas normoxic controls lost less than 0.1% of their body weight (−0.56 ± 1.27 g; Figures 2C,D, 3A,B). After 96-days of exposure, CIH animals gained more (54.4%) body weight (141.78 ± 8.41 g) compared to the normoxic controls (40.1%; 116.49 ± 6.91 g; Figure 3) from their initial weights. Figure 2D also shows that CIH animals gained almost twice the amount of body weight compared to normoxic controls over a 24 h period between days 95 and 96. There was both a significant effect of the duration of exposure and exposure type on body weight (Figures 2C,D) and there was a significant effect of exposure between CIH and normoxic control groups. The CIH group gained more weight overnight than the normoxic group, and this overnight body weight gain increased over the length of exposure (Figure 2D).

### Adipose Tissue Mass Changes

**FIGURE 4:**
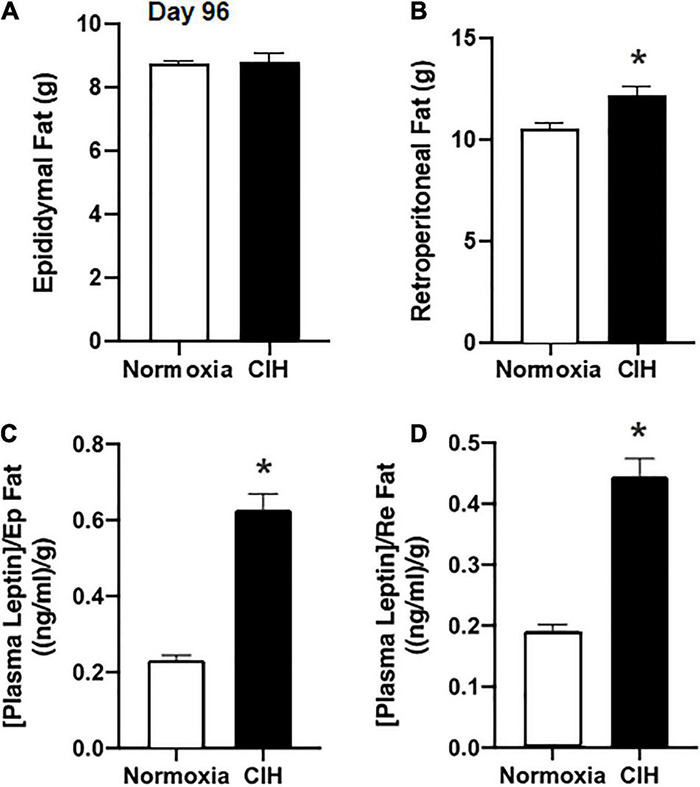
Bar charts summarize epididymal **(A)** and retroperitoneal **(B)** fat pad mass after 96 days of CIH or normoxia exposure. Note that weight change in retroperitoneal (Re) adipose tissue mass was altered after CIH, but that of epididymal (Ep) adipose tissue mass was not altered. **(C,D)** Show plasma leptin levels normalized for Ep and Re adipose tissue mass. Note that in both cases basal plasma leptin levels are elevated when fat pad mass is taken into account. *n* = 16 for each group. Data are shown as mean ± standard error. *p < 0.05.

Epididymal fat pad mass and the retroperitoneal fat pad mass were removed at the completion of the study. Although there were no significant differences in the epididymal fat mass between the CIH and normoxic groups (*p* = 0.4146) (Figure 4A), a significant increase (approximately 15.9%) in the retroperitoneal fat pad mass was found in the CIH group compared to the normoxic group (*p* = 0.0019) (Figure 4B).

### Plasma Leptin Concentrations

Plasma concentrations of leptin during non-fasting conditions following 96 days of CIH were 5.51 ng/ml, while in the normoxia group plasma leptin was 2.03 ± 0.12 ng/ml (*p* < 0.0001). When the plasma leptin levels were normalized to either the epididymal or retroperitoneal fat pad masses in each animal, CIH exposed animals had a significantly (*p* < 0.0001) elevated plasma leptin level compared to the normoxic controls (Figures 4B–D).

### Food Intake in Response to Leptin Injection

**FIGURE 5:**
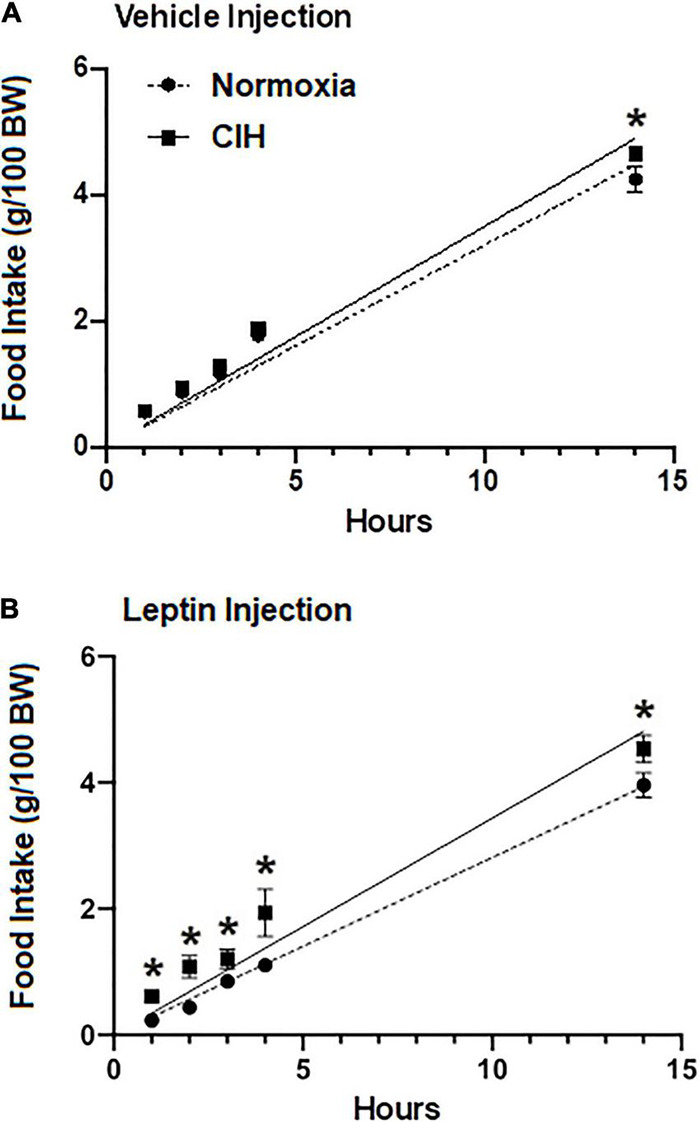
Line graphs showing food intake at 1, 2, 3, and 14 h post-injection (ip) of either 40 ng/kg in 1 ml (range; 14.8–19.1 ng ml) leptin (*n* = 12; *n* = 6 for each normoxic and CIH group) **(A)** or the vehicle (*n* = 11; *n* = 5 for each normoxic and *n* = 6 for CIH group) **(B)**. Note that leptin induces satiety in the normoxic group, but not in the CIH exposed group. Data are presented as mean ± standard error. *p < 0.05.

To investigate whether the chronic elevated leptin level induced by the CIH altered the effect of exogenous injections of leptin on food intake, normoxic controls and CIH exposed animals were subjected to either injections of the vehicle or leptin (Figure 5). In animals exposed to normoxia, food intake 1 h after acute leptin injection was significantly reduced compared to vehicle injected controls (*p* = 0.0003) and compared to CIH exposed animals (*p* = 0.4069) (Figure 5B). Similarly, at 2, 3, 4, and 14 h after the acute leptin injection, food intake was significantly (2 h, *p* = 0.0024; 3 h, *p* = 0.0252; 4 h, *p* = 0.0269; 14 h, *p* = 0.0269) reduced in the normoxic animals compared to the CIH animals (Figure 5B). Both CIH and the normoxic animals had the same food intake following injections of the vehicle (Figure 5A) except at the 14 h period where CIH groups food intake was greater (*p* = 0.0469) than that of the normoxic controls.

### Protein Expression of Leptin Receptors After Chronic Intermittent Hypoxia

**FIGURE 6:**
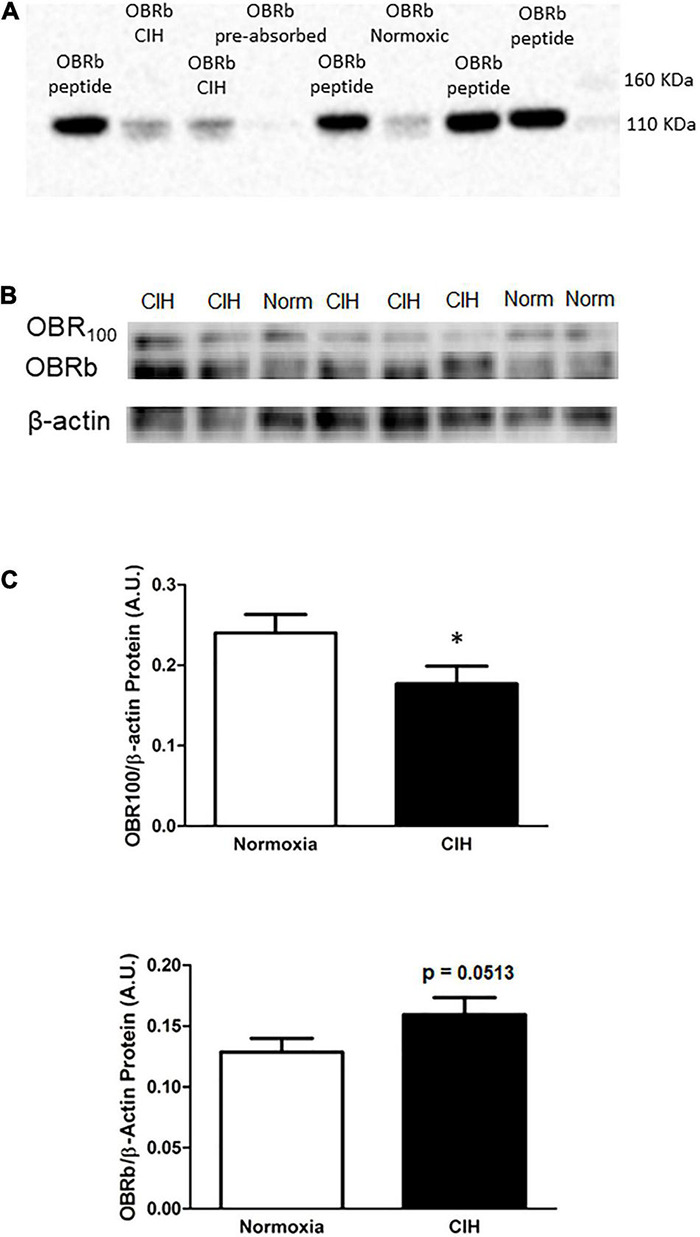
**(A)** Is a representative western blot depicting OBRb/β-actin protein expression in the ARC following exposure to CIH or normoxia, and negative control following pre-absorption of the primary antibody with the corresponding immunizing peptide. Note that the absorption of the antibody (CH14104; Neuromics) with the LepRB/Orb control protein (P14014; Neuromics) did not result in the labeling of the OBRb protein in the ARC. **(B,C)** Representative Western blots **(B)** and bar graphs **(C)** depicting OBR100/β-actin and OBRb/β-actin protein expression in the ARC following CIH exposure. Note that leptin receptor isoform (OBR_100_) thought to be involved in transporting leptin across the blood brain barrier is reduced, whereas the OBRb isoform does not change although there is a trend toward an increase following CIH exposure. Data are shown as mean ± standard error. *n* = 10 per group. *p < 0.05.

As shown in Figures 6B,C, within the ARC, the protein expression of the short form leptin receptor OBR_100_ after CIH was significantly (*p* = 0.0416) reduced compared to normoxic controls (Figures 6B,C). On the other hand, the long-form leptin receptor (OBRb) was not significantly (*p* = 0.0513) elevated in the CIH group compared to normoxia controls, although a trend toward an increase was evident (Figures 6A–C). An example of the absorption control for OBRb is shown in Figure 6A.

### Proteins Associated With Downstream Mediators of Leptin Receptor Signaling Within the Arcuate Nucleus

**FIGURE 7:**
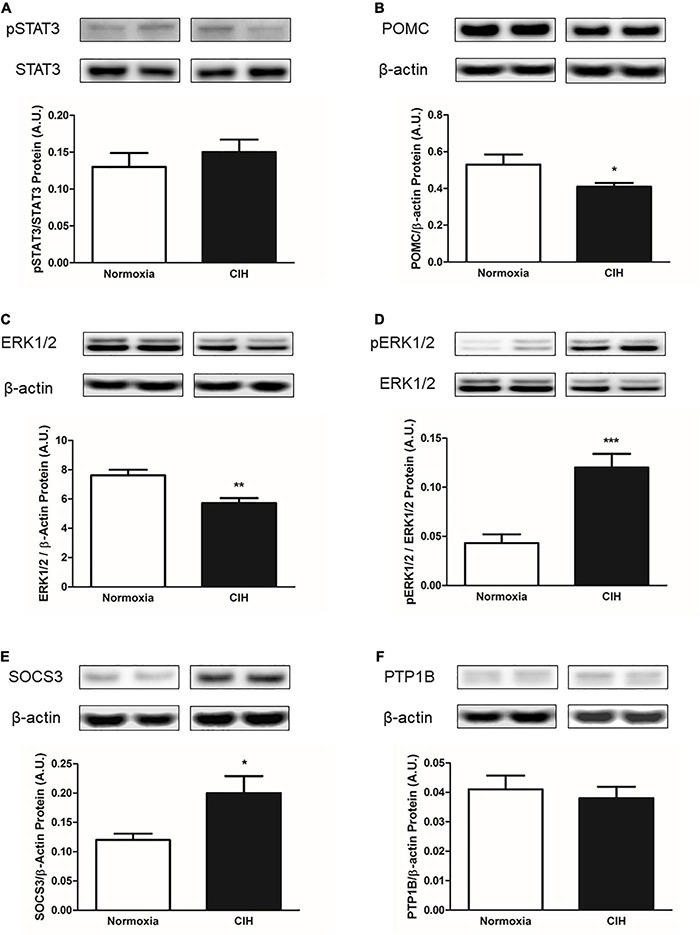
Representative western blots and bar charts depicting pSTAT3/STAT3 **(A)** protein expression, POMC **(B)** protein expression, total ERK1/2 protein expression **(C)**, pERK1/2/ERK1/2 **(D)**, and inhibitors of leptin signaling SOCS3 **(E)** and PTP1B **(F)** protein expressions within the ARC. Note that CIH decreases the expression of the leptin signaling protein POMC and ERK1/2, while increasing the expression of pERK1/2 within the ARC. Also note that CIH increases the expression of SOCS3, but not the PTP1B protein expression after 96 days exposure. Data are presented as mean ± standard error. *p < 0.05, **p < 0.005, and ***p < 0.001. *n* = 10 per group.

Within the ARC, CIH resulted in a trend toward an increase in pSTAT3 protein expression as a function of total STAT3 (*p* = 0.0534) compared to normoxic controls, but neither pSTAT3 nor total STAT3 protein expression (*p* = 0.39) were significantly changed (Figure 7A). Protein expression levels of POMC were found to be significantly less (*p* = 0.0451) in CIH exposed animals compared to the normoxic animals (Figure 7B). Protein expression of total ERK1/2 was significantly less (*p* = 0.0045) in CIH animals compared to that of the normoxic animals (Figure 7C). On the other hand, a significant increase (*p* < 0.0001) in the proportion of ERK1/2 that was phosphorylated was observed in CIH animals compared to normoxic control animals (Figure 7D).

### Protein Markers Associated With Inhibition of Leptin Signaling Within the Arcuate Nucleus

Within ARC, CIH significantly increased (*p* = 0.0221) the expression of SOCS3 protein compared to normoxic controls (Figure 7E). On the other hand, no significant difference (*p* = 0.56) in PTP1B protein expression was observed between the CIH and normoxic animals (Figure 7F).

## Discussion

**FIGURE 8:**
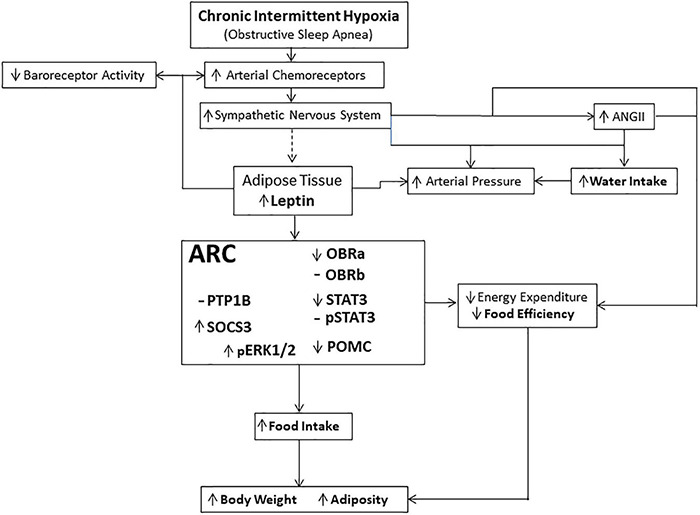
Schematic representation of the physiological pathways involved in mediating the effects of long-term exposure to continuous intermittent hypoxia on leptin and body weight. Dashed line (—) indicates that the link between the activation of the sympathetic nervous system and release of leptin is suggestive based on the evidence of sympathetic innervation of adipocytes and their shrinkage in size during activation, and the data suggesting that plasma leptin concentration is related to adipocyte size (Guerre-Millo, 1997; Jéquier, 2002; Ciriello, 2013; Ciriello and Moreau, 2013). Hypothalamic centers receive and integrate the leptin signal, through activation of leptin receptors, that manifests itself through several effector systems, including the sympathetic nervous system controlling energy expenditure (Guerre-Millo, 1997; Haynes, 2000; Jéquier, 2002).

There are considerable clinical data suggesting that patients that suffer from OSA are overweight or obese (Peppard et al., 2000; Lubrano et al., 2012; Berger and Polotsky, 2018), and it is thought that this excessive weight contributes to the development of OSA (Peppard et al., 2000). However, what remains to be determined is whether OSA itself directly contributes to excessive weight gain (Ong et al., 2013; St-Onge and Shechter, 2015; Gileles-Hillel et al., 2016). This study has provided evidence suggesting that there may exist a reciprocal interaction between OSA and weight gain. These data have demonstrated that CIH, the resulting physiological manifestation of OSA (Dempsey et al., 2010), not only increases food intake after approximately 3.5 months of continuous CIH, but also results in increased circulating levels of leptin. Furthermore, CIH induced changes in the expression of leptin signaling proteins within the ARC that are suggestive of the developing trend toward a leptin resistance (Figure 8). These data suggest that CIH and the resulting increased plasma leptin levels may not only contribute to long-term deleterious effects observed in overweight and obese patients (Phillips and Cistulli, 2006; Gileles-Hillel et al., 2016; Figure 8), but may also impact non-overweight patients with OSA as these patients also display increased circulating levels of leptin and associated cardio-metabolic disorders over time (Pamidi et al., 2012).

We have previously described the acute effects of intermittent hypoxia on body energy balance (Moreau and Ciriello, 2013). Each short-term bout of intermittent hypoxic exposure induced a loss of body weight, which was abrogated during the 24 h period following non-hypoxic conditions, along with reduced locomotion and altered body energy utilization. However, during this time each short-term bout of intermittent hypoxic exposure resulted in an increase in plasma leptin (Moreau and Ciriello, 2013). These earlier observations were supported by the findings in this study of a loss of body weight during the initial bouts of CIH. Given that there is a previously reported reduction (Moreau and Ciriello, 2013), it may be suggested that the initial break-down of adipose tissue contributes to the body weight loss, a finding also suggested by others (Martinez et al., 2008; Drager et al., 2011). However, it should be kept in mind that this may not be the primary mediator of the decreased body weight observed following acute intermittent hypoxia (Moreau and Ciriello, 2013). It is likely that this effect was at least partially due to a potentiation of the peripheral chemoreceptor reflex on sympathetic flow (Ciriello and Moreau, 2012, 2013), consistent with the finding that CIH has long-term effects on sympathetic activity (Zoccal et al., 2007, 2008) and release of angiotensin II (Moreau et al., 2015; Figure 8). These effects are thought to be independent of a change in respiration (Prabhakar et al., 2005, 2009; Dick et al., 2007; Xing and Pilowsky, 2010).

This study has further demonstrated that by approximately 2 weeks of CIH, body weights were similar between the CIH exposed group and the normoxic controls. Interestingly, plasma leptin levels are elevated during this period (Messenger et al., 2012, 2013; Moreau and Ciriello, 2013) and continue to remain elevated as shown in this study after 3.5 months of continuous exposure to intermittent hypoxia during sleep. The body weights of this latter group displayed a modest increase that surpassed that of the normoxic controls. Prolonged exposure to CIH had reversed the initial reductions in body weight observed after short term intermittent hypoxia exposure seen in this study and previous reports (Peppard et al., 2000; Moreau and Ciriello, 2013). Additionally, the elevated plasma leptin level observed at this time did not induce the expected reduction in food intake and an increase in energy expenditure (Figure 8) as the animals ingested greater amounts of food. These data suggest that the animals have become resistant to the effects of circulating leptin. This suggestion is further supported by the lack of a decrease in food intake in response to exogenous administered leptin. However, the gain in body weight in the CIH exposed group may involve several mechanisms, some of which as shown in this study, may include increased food and water intake, decreased food conversion efficiency, decreased locomotor activity, and hypothalamic changes in the expression of proteins associated with regulating some of the physiological effects of leptin. On the other hand, these combined observations are also suggestive of CIH contributing to the induction of a state of leptin resistance (Kalra et al., 1998; Trujillo et al., 2011).

It has previously been demonstrated that leptin resistance is followed by the development of weight gain and hyperphagia (Kalra et al., 1998; Trujillo et al., 2011). It should be kept in mind that similar findings of increased body weight have been reported in patients with chronic sleep disturbances (St-Onge and Shechter, 2015). OSA induction of sleep disturbances with the associated arousals may increase food intake beyond the energy costs of being awake (St-Onge and Shechter, 2015). Overnight decrease in food intake was observed in animals exposed to CIH during the first week compared to normoxic controls. This acute effect on food intake to intermittent hypoxia has previously been reported (Moreau and Ciriello, 2013) and has been observed in humans exposed to chronic hypoxia (Tschop et al., 1998). However, in this study it was found that by the second week of continuous CIH exposure food intake between the CIH and normoxic control groups did not differ, and continual exposure to the intermittent hypoxic stimulus further increased food intake to the point that by 3.5 months of exposure food intake was significantly elevated compared to the amount normoxic controls ingested.

Similarly, food conversion efficiency, an indirect measure of whole-body energy utilization, was reduced in the CIH group over the total exposure period. This is suggestive of decreased body energy utilization. However, this change was not observed in normoxic animals, suggesting their body energy metabolism was not changing in a way concurrent to the CIH animals. Although the long-term effects that food conversion efficiency has on pre-disposition to weight gain is not well understood, a reduction in metabolic function is associated with weight gain in humans (Houmard, 2008). Whether the lack of increase in body energy utilization is due to leptin resistance in the CIH group is not known, but it is possible that given that leptin augments metabolic activities mediated in part by the sympathetic nervous system (Phillips et al., 2000; Harris, 2014; Caron et al., 2018), and plasma leptin levels are elevated following each bout of intermittent hypoxia as found in this study and as previously described (Moreau and Ciriello, 2013), and overall in the CIH group, is further suggestive that long term exposure to intermittent hypoxia contributes to a development of leptin resistance. In support of this suggestion, another measure of leptin resistance was obtained in this study following the acute intraperitoneal injection of leptin into both CIH group and normoxic controls. By a repeated comparison to leptin and vehicle treatment in both groups, it was found that in normoxic animals, acute leptin injections caused a significant reduction in food intake during the first 14 h. However, in the CIH exposed animals, leptin did not induce a satiating effect when compared to vehicle injections. Interestingly, after the 3.5 months of exposure, the amount of epididymal fat pad mass was not altered by the prolonged CIH exposure, whereas the retroperitoneal fat pad mass was significantly increased following the long-term exposure to CIH. These white adipose tissue masses are thought to significantly contribute to plasma leptin levels (Priego et al., 2009; Chusyd et al., 2016). It has been reported that an increase in retroperitoneal fat mass during high fat diets result in a significant decrease in OBR’s mRNA levels (Priego et al., 2009; Chusyd et al., 2016). These effects have been suggested to subsequently contribute to a decrease in leptin sensitivity and a concomitant increase in leptin resistance (Priego et al., 2009; Chusyd et al., 2016). Direct measure of plasma leptin in the CIH animals, showed that there was an almost threefold increase compared to normoxic controls. Furthermore, basal plasma leptin concentrations in the CIH group normalized to epididymal or retroperitoneal fat mass were found to be also about threefold higher than in the normoxic controls. It should be noted that these fat masses are thought to be the largest producer of leptin in the rat (Zheng et al., 1996).

Due to the complex interaction of CIH and this likely leptin resistant state on body weight and fat deposition, it may be suggested that these effects may have impacted these animals after the prolong exposure to CIH as previously suggested (Guo et al., 2013). Although short-term CIH induces anorexigenic effects through increased sympathetic activity (Haynes, 2000; Wolk et al., 2003), evidence indicates that CIH causes a wide range of metabolic pathophysiology including dyslipidemia, high blood pressure and insulin resistance (Polotsky et al., 2003; Zoccal et al., 2007; Drager et al., 2011, 2013; Del Rio et al., 2016), and it has been reported that OSA worsens the metabolic profile of overweight or obese patients (Kono et al., 2007). Thus, the increased weight gain observed in the CIH group may be due to the indirect effects of CIH on these metabolic pathophysiological conditions.

The finding in this study that water intake was increased in the CIH group after long-term exposure was unexpected. Previous studies have demonstrated in OSA patients that the patients often report of waking with a dry mouth and feeling thirsty (Oksenberg et al., 2006; Zhang et al., 2021). The findings that following an overnight bout of intermittent hypoxia plasma levels of both leptin and angiotensin II increased (Moreau et al., 2015) suggest that direct activation of the sympathetic nervous system by leptin (Wolk et al., 2003) may contribute to the feeling of a dry mouth by altering saliva production, while the elevated levels of angiotensin II likely activate central neuronal systems driving thirst (Gutman et al., 1986, 1988).

This study also examined the effect of CIH on leptin receptors expression and some of the known downstream mediators of leptin receptor signaling within the ARC. Following CIH, within the ARC, a decrease was observed in the protein expression of the short-form leptin receptor, OBR_100_. While the precise mechanism surrounding leptin resistance is not understood, it has been suggested that a reduction in OBR_100_, where a reduction in this protein would prevent sufficient transport of leptin across the blood-brain barrier (Banks, 2001) may be involved. It was also observed that the protein expression of the OBRb was not elevated following CIH. It has been suggested that a reduction in OBRb may prevent appropriate leptin signaling (Schwartz et al., 1997). However, the data in this study suggests that due to the decreased transport of leptin across the blood-brain barrier resulting from the decreased OBR_100_ expression, an over expression of OBRb may be occurring in response to the decreased leptin availability. However, this change did not reach our accepted level of statistical significance in this study. From these data, it appears that changes in the expression of the OBR’s may contribute to the development of a leptin resistance, though this may not be the only mechanism driving the leptin resistance in this model. However, it should be kept in mind that these data do not account for the possible changes in affinity for the receptor (Mooradian et al., 2000), or changes in receptor localization (Gan et al., 2012).

Within the ARC, it was observed that CIH animals had no change in the expression of pSTAT3 but had a decreased expression of POMC protein than normoxic control animals. pSTAT3 binds to and regulates POMC gene expression, thus promoting the physiological actions of leptin, including satiety and energy expenditure (Liu et al., 2021). However, it has previously been shown that pSTAT3 increases after short term intermittent hypoxia in the ARC (Moreau and Ciriello, 2013). The primary pathway associated with leptin’s satiety inducing effects is the phosphorylation of STAT3 (Ladyman and Grattan, 2004; Münzberg et al., 2005; Gorska et al., 2010) and the transcription of several genes, such as POMC (Auernhammer et al., 2000). In responses to acute intermittent hypoxia, POMC protein content within ARC was previously reported to increase (Moreau and Ciriello, 2013). Leptin, which is elevated during this period in acute intermittent hypoxia (Moreau and Ciriello, 2013) has been shown to increase the activity of ARC POMC-containing neurons (Wang et al., 2008), while increased POMC activity induces hypophagia (Zhang et al., 2021). Acute intermittent hypoxia induced both an increase in the ARC POMC, as well as hypophagia (Moreau and Ciriello, 2013). However, in contrast, this study has shown that long term intermittent hypoxia appears to induce the opposite effects. Consistent with this finding, a reduction in POMC within the ARC has been shown to be associated with hyperphagia (Richard et al., 2011). This may account for the increased food intake, decreased food conversion efficiency and may have contributed to the overall increase in body weight observed in the CIH animals (Liu et al., 2021).

Furthermore, within the ARC, SOCS3 protein expression was found to be greater in the CIH group than in the normoxic controls. An increase in SOCS3 would prevent activation of the leptin receptor signaling cascade (Bjørbaek et al., 1998; Bjorbak et al., 2000), and preventing downstream activation of factors associated with the effects of leptin, such as POMC (Banks et al., 2000). SOCS3 overexpression has previously been shown to occur in leptin resistant states, and experimentally can increase food intake (Bjørbaek et al., 1998; Reed et al., 2010). These changes are concomitant with a lower protein amount of ERK1/2 and increased phosphorylation of ERK1/2 in the ARC in the CIH group (Myers et al., 2010). Interestingly, no changes were observed with the protein expression of PTP 1 B as this protein plays an important role in limiting the extent of leptin’s action (Bence et al., 2006).

Taken together, these data have demonstrated for the first time that long-term exposure to CIH is associated with an increase in energy balance which may result from the development of a leptin resistant state. This pathophysiological state is associated with a dysregulation of body weight involving a lack of balance between energy intake and expenditure (Myers et al., 2008, 2010; Morris et al., 2010) and is associated with an increased protein expression of SOCS3, and with a concomitant reduction in POMC protein expression within the ARC. In conclusion, these findings suggest a possible link between non-obese individuals exposed to CIH, such as those experiencing OSA, and the development of obesity and related metabolic disorders.

## Materials and Methods

### General Animal Procedures

Experiments were done in 32 adult male Sprague-Dawley rats (Charles River Canada, St. Constant, Canada) weighing 290.1 ± 2.9 g individually housed in a room maintained at a temperature of 22°C and 60% relative humidity. All animals had access to food and water *ad libitum* in 12 h light/dark cycle conditions. Animals were randomly assigned to either CIH (*n* = 16) or normoxia (*n* = 16) groups and exposed to the corresponding conditions for 96 consecutive days. All experimental procedures and handling of animals were done in accordance with the guidelines on the use and care of laboratory animals as set by the Canadian Council on Animal Care and approved by the Animal Care Committee at the University of Western Ontario (AUP 2008-03-04-5 and AUP-2008-04-8).

### Chronic Intermittent Hypoxia and Normoxic Exposures

Animals were exposed to CIH or normoxic conditions as previously described (Messenger et al., 2012, 2013; Messenger and Ciriello, 2013; Moreau and Ciriello, 2013). The animals were exposed to 8 h CIH or normoxic stimuli each day (0900–1,700 h) of the experimental period during their sleep time, as rodents are nocturnal. The animals were preconditioned to the experimental chambers and tubes for at least 3 days prior to the start of the study. In brief, animals were placed in the chambers, each chamber consisting of four plexiglass tubes (10 cm diameter by 35 cm length) and a zero-pressure escape valve. The animals were not restrained and were able to freely move within the tubes. For CIH-exposed animals, computer regulated solenoid valves altered the input of N_2_ or room air to generate CIH conditions. Animals were exposed to 80 s hypoxia (6.5% O_2_) followed by 120 s normoxia (Messenger et al., 2012, 2013; Messenger and Ciriello, 2013; Moreau, 2013; Moreau and Ciriello, 2013). The levels of O_2_ and CO_2_ were continuously monitored by sensors within the chamber, which relayed information back to the computer to ensure proper cycling. Conditions within the chamber were isobaric (770 ± 11 mmHg) and eucapnic (<0.1% CO_2_). Normoxic animals were treated similarly, but only exposed to room air within the chambers.

### Measurement of Body Weight, and Food and Water Intake

Throughout the study (on days 1, 14, and 96), animals were weighed immediately before and after CIH or normoxic exposure and immediately before the next exposure on the following day. These values were used to calculate body weight changes during the exposure period, overnight body weight gain and 24 h body weight change. Additionally, food and water intake were measured over the 16 h immediately following CIH or normoxic exposure on each day and normalized to each animal’s body weight for statistical comparisons.

### Locomotion Assay

Immediately following CIH or normoxic exposure on days 91 and 92, animals were returned to their home cages and were allowed access to food and water *ad libitum*. Thirty minutes into the dark (active) cycle (1,930 h), animals were placed into large cages (60 × 40 cm) with a floor 4 × 5 grid system in the dark (red light on). During a 5-min period, the number of crosses of the midline by an animal was determined by two independent observers blinded to their exposure regime to determine horizontal locomotion (Moreau and Ciriello, 2013). Simultaneously, vertical locomotion was determined by the number of rearing events. An average value was then calculated from these two independent observations for both horizontal and vertical locomotion (Moreau and Ciriello, 2013).

### Leptin Injections

Following CIH or normoxic exposure on (days 93), animals were placed into their home cages with *ad libitum* access to food and water. At the beginning of the dark cycle (1,900 h), animals from the CIH or normoxic groups were randomly assigned to receive either a vehicle or a physiological dose of leptin (0.04 mg/kg) carrier-free recombinant rat leptin (598-LP; R&D Systems, Minneapolis, MN) dissolved 1 ml in 20 mM Tris HCl (pH = 8.0), injection (ip). Food intake measurements were taken at 1, 2, 3, and 14 h after the leptin injection. Animals were exposed to the previous CIH or normoxic conditions for the next 2 days prior to euthanasia.

### Plasma Collection and Enzyme Immunoassays

Following the last day of exposure (day 96), animals were immediately (within 15–20 min following exposure) euthanized under equithesin anesthesia (0.3 ml/100 g body weight; ip) (Moreau and Ciriello, 2013). Blood samples were collected by cardiac puncture in the presence of 7% ethylenediaminetetraacetic acid at a volume of 10 μl/ml blood. This blood was immediately centrifuged at 10,000 RPM for 10 min at 4°C to isolate the aqueous plasma. This aqueous plasma phase was removed and stored frozen at −80°C until analyzed for hormone content. Plasma samples were analyzed using enzyme immunoassay for rat leptin (Enzo Life Sciences; Farmingdale, NY) according to the manufacturer instructions. Enzyme immunoassay plates were read on a SpectraMax M5 plate reader using SoftMax Pro v.5 microplate analysis software (Molecular Devices; Sunnyvale, CA) (Messenger et al., 2012).

### Tissue Collection and Preparation

Immediately after euthanasia, the brains were immediately removed and frozen at −80°C. Sections of the forebrain (500 μm) were cut in a cryostat. The region of the ARC was identified (approximately 6.5 mm rostral to the interaural line; Paxinos and Watson, 1986) in the sections and using a circular 1 mm (internal diameter) micro-punch tool, 500 μm thick punch-outs of the ARC, bilaterally, were taken and immediately homogenized in cold radioimmunoprecipitation assay buffer (50 mM Tris, 150 mM NaCl, 1% Triton-X 100, 0.25% sodium deoxycholate, 1 mM NaF, 1 mM sodium orthovanadate, 25 mM β-glycerophosphate) with protease inhibitor cocktail (Roche Applied Science; Laval, QC) by an electric homogenizer (VWR International; Radnor, PA). Homogenates were then sonicated over three passages for 15 s each on ice (55%; Sonic Dimembrator Model 150; Fisher Scientific). Samples were then rotated for 10 min at 4°C and centrifuged at 4°C for 20 min at 14,000 RPM. Protein content of homogenates was quantified using the Bio-Rad Dc protein assay kit (Bio-Rad Laboratories; Hercules, CA). Protein samples were added to 25% sample buffer and 10% reducing buffer (Life Technologies; Burlington, ON) and water to a standard protein concentration of 1.67 mg/ml (Messenger and Ciriello, 2013; Moreau and Ciriello, 2013).

### Western Blots

Electrophoresis was carried out using a 10% discontinuous polyacrylamide Bis-Tris gel (Life Technologies; Burlington, ON), followed by standard protein immunoblotting techniques (Messenger et al., 2013; Moreau, 2013; Moreau and Ciriello, 2013). For each animal, 25 μg of protein of each sample was loaded. Electrophoresis was carried out at 200 V and terminated when the dye front reached the bottom of the gel. Proteins were transferred to a polyvinylidene fluoride membrane using a wet transfer method in the presence of methanol and SDS (50 mM Tris, 40 mM glycine, 0.3% SDS, 20% methanol) and wet transfer apparatus (Mini Trans-Blot Electrophoretic Transfer Cell; Bio- Rad Laboratories; Hercules, CA) at 100 V for 2 h. After transfer, the membrane was washed in Tris-buffered saline + Tween-20 (TBST; 20 mM Tris-HCl, 0.5 M NaCl, 0.1% Tween-20; pH 8.0) blocked for 1 h with 5% skim milk made in TBST buffer at room temperature. The membrane was then incubated with primary antibodies diluted in skim milk over night at 4°C. The following day, the membrane was washed with TBST before being incubated with horseradish peroxidase-conjugated secondary antibodies-specific to the appropriate host of the primary antibody being analyzed, for 1 h at room temperature. For detection, the membrane was washed with TBST, followed by distilled water, and then stained using a horseradish peroxidase substrate enhanced chemiluminescence system (Luminata Forte, EMD Millipore; Billerica, MA). Blots were visualized using a VersaDoc imaging system (Bio-Rad Laboratories; Hercules, CA) and analyzed using ImageLab v.3.0 (Bio-Rad Laboratories; Hercules, CA). Details and examples of the Western blots have been previously reported (Moreau, 2013).

### Antibodies

For Western blots the following antibodies were used: rabbit anti-β-actin-HRP (1:50,000; A3854, Sigma-Aldrich; St. Louis MO), rabbit anti-OBR (1:1,000; OBR12-A, Alpha Diagnostics International; San Antonio, TX), chicken anti-OBRB (1:5,000; CH14104, Neuromics; Edina, MN), monoclonal anti-OB-R (sc-8391) Santa Cruz), rabbit anti-STAT3 (1:2,000; #9132, Cell Signaling; Boston, MA), rabbit anti-pSTAT3 (Tyr705) (1:1,000; #9131, Cell Signaling; Boston, MA), rabbit anti-ERK1/2 (1:2,000; #9102, Cell Signaling; Boston, MA), rabbit anti-pERK1/2 (Thr 202/Tyr204) (1:1,000; #9101, Cell Signaling; Boston, MA), rabbit anti-POMC (1:2,000; RB-08-0013, RayBiotech; Norcross GA), rabbit anti-SOCS3 (1:1,000; ab16030, Abcam; Cambridge, MA), goat anti-PTP1B (1:500; sc-1718, Santa Cruz Biotechnology; Dallas, TX), donkey anti-rabbit IgG-HRP (1:10,000; 711-035-152, Jackson Immunoresearch; West Grove, PA), donkey anti-chicken IgY-HRP (1:10,000; 703-035-155, Jackson Immunoresearch; West Grove, PA), donkey anti-goat IgG-HRP (1:10,000; 705-035-003, Jackson Immunoresearch; West Grove, PA). Negative controls were performed by omission of primary antibody and/or through the absorption of the primary antibodies with an excess of the corresponding immunizing peptides for the OBR’s (CH14104, for the antibody specific for detecting OBR and P14014, the LepRB/Orb control blocking protein, Neuromics; sc-8391 for detecting long and short form of the OBR’s and sc-8391 blocking peptide, Santa Cruz) did not result in the detection of the OBR proteins in the ARC (Figure 6).

### Statistics and Analysis

Differences in physiological variables between and within CIH and normoxic groups between days 0–1, 13–14, and 95–96 were determined by two-way ANOVA with a repeated measure between the exposure time groups and the physiological variable measured, followed by a Bonferroni *post hoc* analysis. Plasma leptin concentrations, Western blot analyses and fat pad mass between CIH and normoxic groups were compared using an unpaired, two-tailed *t*-test. For leptin injection assays, a two way-ANOVA followed by a paired two-tailed *t*-test was used to compare vehicle injection to leptin injection within CIH and normoxic groups. For all statistical analyses, a *p*-value < 0.05 was taken to indicate statistical significance. All values were expressed as mean ± standard error. All charts were made using GraphPad Prism v.5 graphing software (GraphPad Software; La Jolla, CA).

## Data Availability Statement

The original contributions presented in the study are included in the article, further inquiries can be directed to the corresponding author/s.

## Ethics Statement

The animal study was reviewed and approved by the University of Western Ontario (AUP 2008-03-04-5 and AUP-2008-04-8).

## Author Contributions

JC performed some of the studies, wrote, edited, and approved the final manuscript. JM performed some of the studies and provided a draft manuscript. MC contributed to the data analysis, edited, and finalized the manuscript for publication. RM completed the analysis, illustrations, and reviewed the manuscript. All authors contributed to the article and approved the submitted version.

## Conflict of Interest

The authors declare that the research was conducted in the absence of any commercial or financial relationships that could be construed as a potential conflict of interest.

## Publisher’s Note

All claims expressed in this article are solely those of the authors and do not necessarily represent those of their affiliated organizations, or those of the publisher, the editors and the reviewers. Any product that may be evaluated in this article, or claim that may be made by its manufacturer, is not guaranteed or endorsed by the publisher.
